# The choreography of chromatin in RNA polymerase III regulation

**DOI:** 10.1042/BST20230770

**Published:** 2024-04-26

**Authors:** Maria Elize van Breugel, Alan Gerber, Fred van Leeuwen

**Affiliations:** 1 Division of Gene Regulation, Netherlands Cancer Institute, Amsterdam 1066 CX, The Netherlands; 2 Department of Neurosurgery, Amsterdam UMC Location Vrije Universiteit Amsterdam, Amsterdam 1081HV, The Netherlands; 3 Cancer Center Amsterdam, Cancer Biology, Amsterdam 1081HV, The Netherlands; 4 Department of Medical Biology, Amsterdam UMC, University of Amsterdam, Amsterdam 1105 AZ, The Netherlands

**Keywords:** chromatin, RNA polymerase III, transcription, tRNA

## Abstract

Regulation of eukaryotic gene expression involves a dynamic interplay between the core transcriptional machinery, transcription factors, and chromatin organization and modification. While this applies to transcription by all RNA polymerase complexes, RNA polymerase III (RNAPIII) seems to be atypical with respect to its mechanisms of regulation. One distinctive feature of most RNAPIII transcribed genes is that they are devoid of nucleosomes, which relates to the high levels of transcription. Moreover, most of the regulatory sequences are not outside but within the transcribed open chromatin regions. Yet, several lines of evidence suggest that chromatin factors affect RNAPIII dynamics and activity and that gene sequence alone does not explain the observed regulation of RNAPIII. Here we discuss the role of chromatin modification and organization of RNAPIII transcribed genes and how they interact with the core transcriptional RNAPIII machinery and regulatory DNA elements in and around the transcribed genes.

## Introduction

**Figure 1.**
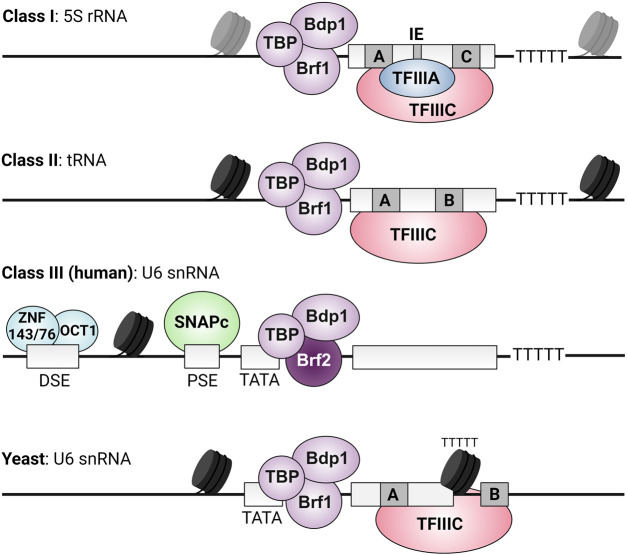
Different classes of RNAPIII-transcribed genes. Class I is composed of the 5S rRNA gene. The internal control regions (ICRs) harbor an A-box, intermediate element (IE) and a C-box. Six-subunit TFIIIC (yeast Tfc1, 3, 4, 6, 7, 8; human orthologues TFIIIC63, 220, 102, 110, 35, 90) is recruited through TFIIIA (yeast PZF1; human orthologue GTF3A). TFIIIC recruits TFIIIB (Brf1, Bdp1 and TBP) [168,169]. Class II RNAPIII-transcribed genes include tRNA genes, adenovirus VA RNA genes, SINE elements and others. The ICRs harbor an A-box and B-box which are recognized by TFIIIC, followed by TFIIIB recruitment [15,16]. Class III is composed of the U6 snRNA gene (except yeast), 7SL/7SK RNA genes and others. Transcription of the human U6 snRNA gene is initiated by binding of SNAPc to the proximal sequence element (PSE). This is stimulated by RNAPII transcription factors STAF (ZNF143 or ZNF76) and Oct1 that bind to the enhancer-like distal sequence element (DSE) [57,170]. A positioned nucleosome between the DSE and PSE allows efficient recruitment of SNAPc [171]. SNAPc facilitates TFIIIB assembly which contains the Brf1 paralogue Brf2. The yeast U6 snRNA gene (*SNR6*) is not considered a class I or II promoter and is also different from vertebrate class III promoters [172]. *SNR6* contains an upstream TATA box, an intragenic A-box and extragenic B-box downstream the terminator sequence. A positioned nucleosome brings the A- and B-box in close proximity which allows TFIIIC binding [173]. Nucleosomes are depicted as black cylinders. The organization of nucleosomes at class I genes is not well-understood, as indicated with semi-transparent nucleosomes.

Chromatin is the complex of DNA and histones in the nucleus. It helps organizing the genome but also provides many opportunities for regulation of genome transactions such as replication, transcription, and repair. Indeed, DNA methylation, histone post-translational modifications such as acetylation and methylation, and interactions with non-histone proteins such as transcription factors and chromatin remodelers all affect gene transcription by RNA polymerase II (RNAPII). While the role of chromatin in RNAPII regulation has been extensively studied [1–4], our knowledge on the role of chromatin in the regulation on RNA polymerase III (RNAPIII) is far from complete [5–10]. RNAPIII transcribes several short non-coding RNAs in eukaryotes such as *5S rRNA* (class I), tRNA genes (class II) and the *U6 snRNA* (class III). In mammalian cells, the repertoire of RNAPIII is extended to adenovirus VA genes, 7SL and 7SK RNA, Short Interspersed Nuclear Elements (SINEs) and several other small RNAs [11–14]. Recruitment of RNAPIII is directed by the general initiation factor TFIIIB which is positioned upstream of the transcription start site (TSS) by the sequence-specific general transcription factors TFIIIA (specific to class I), TFIIIC (class I and II) and SNAPc (specific to class III), see Figure 1 [15,16]. Since the majority of RNAPIII-transcribed genes is made up by the class II tRNA genes, which include ∼275 loci in the budding yeast genome and over ∼400 loci in the human genome [17], regulation of class II tRNA genes will be the focus of this review. The general transcription factor TFIIIC recognizes conserved sequence elements within the tRNA gene-body sequence. However, tRNA genes with identical gene-body sequences can be differentially regulated at different genomic loci, for reasons that are still not well understood [18–22]. For example, when the demand for tRNA molecules is low, tRNA gene transcription is repressed by Maf1, a factor that directly interacts with RNAPIII and prevents its recruitment to TFIIIB-bound tRNA genes, but without interfering with the catalytic activity of RNAPIII itself [23–26]. The response of tRNA genes to Maf1 varies among individual tRNA genes [18,27]. In addition, tRNA genes are known to be expressed in a tissue- and cell-type specific manner that cannot be explained by the known regulatory DNA sequences [28–30]. Together, these observations suggest that additional mechanisms of regulation are involved, such as proximal or distal regulatory DNA elements, three-dimensional genome organization and folding, or chromatin-structure based mechanisms.

In this mini-review, we will discuss the role of chromatin in the regulation of RNAPIII transcription with a focus on tRNA genes and how this could contribute to (differential) tRNA gene regulation. The role of chromatin is discussed in the context of three layers involved in RNAPIII regulation. First, the basic RNAPIII transcription machinery and how it recognizes the information embedded within and flanking the gene body are considered. Second, we will discuss the dynamic chromatin context of tRNA genes, including nucleosome positioning, histone modifications and other chromatin-associated factors. Finally, we will discuss the role of the genomic context on RNAPIII regulation with a focus on short-distance interactions such as neighboring genes and long-distance 3D genome organization.

## The basic RNAPIII transcription machinery

### Transcription initiation

In contrast with RNAPII-transcribed genes, which require promoter sequences upstream of the gene body or coding region, tRNA genes are self-contained elements that harbor intragenic promoter sequences [31,32]. These internal control elements, known as A- and B-boxes, are binding sites for the six-subunit assembly factor TFIIIC [33,34]. Optimal binding of the general transcription factor TFIIIC occurs when the A- and B-box are spaced by 30–60 bp [35]. Once TFIIIC is bound, it promotes assembly of the transcription initiation factor TFIIIB ∼50 bp upstream of the TSS of the tRNA gene through interactions between TFIIIC subunit Tfc4 (TFIIIC102 in human) and Brf1 as an initial recruitment step, followed by TBP and Bdp1 binding in a stepwise mechanism [36–39]. TFIIIB is composed of TBP (TATA-binding protein), Brf1 (B-related factor) and Bdp1 (B-double prime) and recruits RNAPIII via multiple contacts between Brf1, TBP and the C34/hRPC39 RNAPIII subunit [37,40–43].

It has long been thought that only the internal control regions contribute to promoter strength of tRNA genes. However, early *in vitro* studies in the silk worm *Bombyx mori* suggested that upstream sequences are responsible for distinct transcriptional properties of tRNA genes [44]. Subsequently, it was found that the TATA-binding protein TBP and the presence of an upstream TATA-like element are key determinants in differential transcription of a subset of tRNA genes in silk worms and *Drosophila* cells, but also in the fission yeast *Schizosaccharomyces pombe* and the plant *Arabidopsis thaliana* [45–48]. In the budding yeast *Saccharomyces cerevisiae*, a TATA-like element is found upstream ∼30% of tRNA genes present in the genome [49]. In contrast with plant and fission yeast, TFIIIB assembly in budding yeast does not strongly rely on TBP-TATA interactions but is instead driven by TFIIIC contacts [50]. The TATA-like sequence might however affect the efficiency of transcription and TSS selection. Additionally, a yeast tDNA-deletion library showed that strains with impaired growth were enriched for the presence of a TATA-like motif, suggesting that this motif may contribute to differential tDNA regulation [20].

Interestingly, a TATA-like element provides the ability to transcribe tRNA genes and the *SNR6* gene of *S. cerevisiae* in a TFIIIC-independent manner *in vitro* using a minimal system of TFIIIB and RNAPIII [51,52]. In contrast, longer transcripts and genes lacking a TATA-like element rely on TFIIIC for efficient RNAPIII transcription *in vitro*, and upon *in vitro* chromatin reconstitution, TFIIIC is required for RNAPIII transcription [53–55]. TFIIIC-independent transcription of RNAPIII-transcribed genes has not been observed *in vivo*, possibly because TFIIIC is required for efficient transcription due to its chromatin organizing activity to relieve the repressed chromatin state [54,56].

Besides the TATA-like element at position -42 at tRNA genes in budding yeast, genome-wide analysis of 5′-flanking regions of tRNA genes revealed additional conserved sequence patterns around positions -53, -30 and -13 [49]. Variants of these patterns may contribute to differential regulation of tRNA genes. Indeed, *in vitro* transcription assays showed differential transcriptional output within tRNA gene families, which correlated with the number of conserved upstream motifs [49]. Another example of upstream regulatory sequences is the involvement of a distal sequence element in tissue-specific expression of the selenocysteine tRNA in metazoans [57,58].

### Transcription termination

Efficient termination of RNAPIII is important for transcript release, reinitiation for multiple rounds of transcription, and to prevent read-through transcription [59,60]. Termination of RNAPIII has long been considered to be solely facilitated by a simple consensus sequence of four or more thymidine residues, called the T-stretch (reviewed in [61–63]). The budding yeast Rpc37-53 RNAPIII subunit-heterodimer in elongating RNAPIII recognizes the termination sequence and sensitizes termination by weakening of the RNA:DNA hybrid [64,65]. While in budding yeast >97% of tRNA genes contain a canonical terminator sequence within the first 15 bp of the 3′-flanking region, only 50–60% of human tRNA genes display a T-stretch within this region [66]. This suggests that additional non-canonical terminators must exist.

Efficient RNAPIII termination is not only facilitated by recognition of the T-stretch, but also depends on the sequence context of the T-stretch [67] and multiple accessory proteins [68,69]. For example, Sen1 (senataxin), a conserved DNA/RNA helicase, is recruited to tRNA genes and 5S rRNA genes in fission yeast to promote efficient RNAPIII termination [68]. Similarly, Sen1 also plays a role in RNAPIII termination in budding yeast [69]. This function relies on the interaction between the N-terminal domain of Sen1 with RNAPIII. Especially at tRNA genes that contain a weak termination signal, Sen1 functions as a fail-safe mechanism to ensure efficient termination and prevent read-through transcription [69].

### Transcription reinitiation

RNAPIII is known for its high reinitiation efficiency to produce high levels of noncoding RNAs. RNAPIII reinitiation is likely coupled to termination in a process called facilitated recycling [70]. For example, a peptide nucleic acid roadblock downstream the terminator sequence reduced RNAPIII reinitiation *in vitro* [71]. RNAPIII recycling is not restricted to small sized templates, but is was also shown on the 522-bp-long *SCR1* gene [72]. Recycling of RNAPIII can take place in a TFIIIC-independent manner on short transcripts (<100 bp) in a reconstituted transcription system from budding yeast using recombinant TBP and Brf1 and a crude extract of Bdp1. However, recycling on longer transcripts (>300 bp) requires TFIIIC [53]. Interestingly, recombinant human Maf1 is unable to repress recycling RNAPIII *in vitro* while it can efficiently repress free RNAPIII*,* indicating that effective tRNA gene repression by Maf1 in cells might require perturbing the facilitated recycling [25]. Details on facilitated recycling of RNAPIII and how it is regulated *in vivo* are currently poorly understood [59]. If recycling takes place *in vivo*, additional chromatin-based factors and mechanisms, such as passage of RNAPII, could possibly interfere with RNAPIII recycling and facilitate Maf1-mediated repression [73,74].

## The dynamic chromatin context

**Figure 2.**
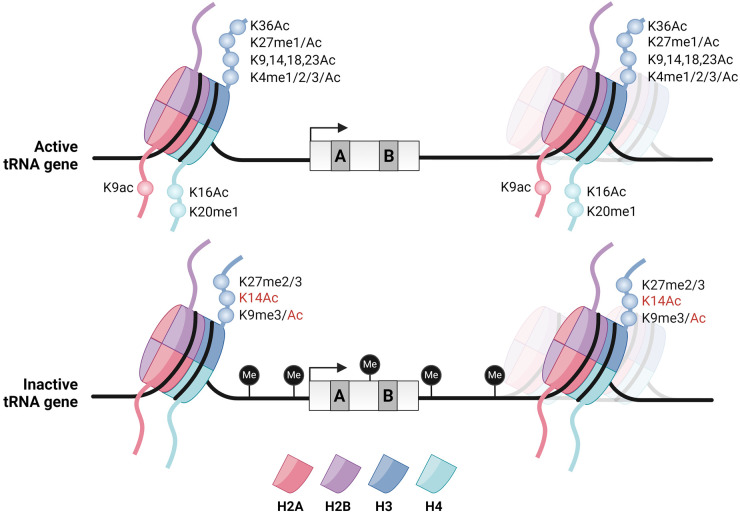
Chromatin modifications at active and inactive tRNA genes. tRNA genes are flanked by upstream and downstream nucleosomes. The upstream nucleosome is located ∼140 bp upstream of the TSS while the downstream nucleosome can have variable positions. Nucleosomes at active tRNA genes are marked with several histone modifications such as acetylation and methylation of histone H3, H4 and H2A. In contrast, the majority of histone modifications are lost at inactive tRNA genes. H3K14Ac and H3K9Ac, usually associated with active tRNA genes are found on a subset of inactive tRNA genes (indicated with red text). Additionally, DNA methylation likely occurs more frequently at inactive tRNA genes as RNAPIII occupancy is anti-correlated with CpG methylation and TFIIIC binding is impaired by DNA methylation [7,12,127,128,136–138].

How transcription by RNAPII is regulated by nucleosome remodeling has been extensively studied [75]. In contrast with protein-coding genes, RNAPIII-transcribed class II genes are considered to be devoid of nucleosomes [76–81]. Nonetheless, chromatin dynamics also play a role in RNAPIII regulation. Here we consider three different classes of chromatin remodeling: nucleosome remodeling, histone variant assembly, and histone modification (Figure 2).

### Nucleosome-positioning

Generally, nucleosomes form a barrier for access to the DNA template by transcription factors and RNA polymerases. Opening up chromatin requires the activity of pioneer transcription factors, histone octamer repositioning or eviction by nucleosome remodelers, or a combination of both. Class II tRNA genes are found in regions largely devoid of nucleosomes [76–81]. The majority of tRNA genes in yeast (>80%) contain a strongly positioned nucleosome ∼140 bp upstream of the TSS while the position of the nucleosome downstream the transcription termination site is variable [77]. While most tRNA genes in budding yeast are devoid of nucleosomes, one study suggests that about half of all tRNA genes in budding yeast are covered by a single highly unstable nucleosome [82]. Upon nutrient starvation in budding yeast, a condition that leads to tRNA gene repression, nucleosomes are remodeled on a fraction of tRNA genes. In addition, nucleosome and RNAPIII occupancy show an inverse correlation, suggesting that nucleosome remodeling is required for efficient RNAPIII repression or vice versa [77]. Corroborating this, active tRNA genes are accessible and contain a well-positioned -1 nucleosome (−150 bp TSS) and +1 nucleosome (+220 bp TSS) and upstream and downstream nucleosome arrays in human cells. In contrast, inactive tRNA genes show low accessibility [76]. However, nucleosome occupancy is commonly defined by the protection of ∼150 bp of DNA from micrococcal nuclease. *In vivo*, other protein-DNA complexes may protect regions of similar sizes, which appears to be the case at tRNA genes bound by TFIIIB-TFIIIC [83]. As a consequence, increased ‘nucleosome occupancy’ in unfavorable conditions could also reflect a stronger association of TFIIIC with tRNA genes [84].

### Nucleosome remodelers

What keeps tRNA genes in an open, nucleosome depleted configuration? Evidence for a first level of regulation comes from *in vitro* transcription studies using chromatinized templates. In such studies it was found that purified human TFIIIC can bind to the A- and B-box of a tRNA gene within a chromatin template and relieve nucleosome-mediated repression [54,85,86]. Interestingly, *in vivo*, human TFIIIC remains bound at tRNA genes even in highly compacted metaphase chromosomes, possibly bookmarking these genes for their rapid reactivation following mitosis [87,88]. Moreover, TFIIIC was found to contain intrinsic histone acetyltransferase (HATs) activity and recruit P300, which might further enhance the chromatin organizing activity of TFIIIC [85,89]. In support of this idea, binding of TFIIIC to Alu elements in human cells has been found to promote histone acetylation at the bound Alu elements [90]. However, P300 supports the interaction of TFIIIC with DNA also independent of its catalytic activity and has a positive effect on histone-free templates, suggesting functions beyond histone acetylation [89]. Beyond P300, TFIIIC itself has been reported to interact with several other factors that can support its role in modifying the chromatin state of tRNA genes. Among these factors are CTCF, condensin II, and several chromatin remodelers [90–93]. One of the TFIIIC-interaction partners in mouse and human cells is SMARCAD1 [92]. However, this chromatin remodeler is not enriched at active tRNA genes and seems dispensable for the role of TFIIIC in RNAPIII transcription. Additionally, independent of its role in RNAPIII regulation, TFIIIC has been proposed to have chromatin organizing properties via its interactions with so-called ETC sites, genomic loci that contain extra TFIIIC and lack TFIIIB and RNAPIII [13,94–98].

Several chromatin remodeling factors have been associated with tRNA genes. The small non-histone protein Nhp6 is a high-mobility group protein that binds and remodels nucleosomes by recruitment of chromatin remodeling complexes. In budding yeast, Nhp6, encoded by the two genes *NHP6A* and *NHP6B*, occupies tRNA genes and the *SNR6* gene [99]. The two genes were identified as high-copy suppressors of a mutant form of the *SNR6* gene that carries a 2-bp deletion in the B-box (*snr6Δ2*) [100]. Vice versa, overexpression of *SNR6* suppressed the growth defect observed in the *nhp6a/b* double knock-out [101]. These studies suggest that Nhp6 is a transcriptional activator of the *SNR6* gene where it promotes correct start site selection of TFIIIC and TFIIIB [102]. Nhp6 plays a similar role at tRNA genes where Nhp6a stabilizes the interaction of TFIIIC with box A and also limits TFIIIB to its most upstream site [103]. Interestingly, not all tRNA genes require Nhp6a and/or Nhp6b for initiation site fidelity and even genes with identical coding sequences genes show a different dependency. This suggests that sequences upstream of the TSS might play a role in the Nhp6 dependency [103]. Indeed, expression of a tRNA gene preceded by a suboptimal TFIIIB binding site was strongly dependent on Nhp6 *in vivo*, leading to the suggestion that Nhp6 selectively activates transcription of tRNA genes that have suboptimal TFIIIB binding sites [99]. However, studies of the *SUP4* tRNA gene in yeast suggest that Nhp6, and the FACT complex that it recruits, also have repressive activities [104].

Another chromatin remodeler found at tRNA genes is the RSC complex. Several RSC subunits are enriched at RNAPIII-transcribed genes. Notably, RSC occupancy varies across tRNA genes [77,105]. In yeast, the RSC subunits Sth1, Rsc1 and Rsc4 have been shown to directly interact with the RNAPIII subunit Rpo31 [106]. However, the interaction does not depend on DNA, which suggests that RNAPIII and RSC interact in a chromatin-context independent manner. A *rsc4* C-terminal mutant *rsc4-Δ4*, which abolishes the interaction between Sth1 and Rpo31, did not affect transcription of tRNA genes [106]. This suggests that interactions between RSC and RNAPIII are not critical for efficient transcription of tRNA genes. However, the *rsc4-Δ4* mutant did show increased nucleosome occupancy at tRNA genes [77] and loss of Sth1 leads to a reduction in RNAPIII occupancy and gain in nucleosome density at the *SCR1* gene and tRNA genes [107]. Therefore, RSC might maintains RNAPIII-transcribed genes nucleosome free to facilitate transcription.

In contrast with RSC, which is mainly active downstream of the TSS, the chromatin remodeler ISW2 is found upstream of tRNA genes [77]. Deletion of a tRNA gene significantly reduces Isw2 binding which suggests that the RNAPIII transcription machinery is required for Isw2 enrichment [108]. This is in agreement with the observation that the N-terminal domain of TFIIIB subunit Bdp1 is required for binding of the Isw2 complex to tRNA genes [109]. Isw2-occupied tRNA genes are also enriched for Isw1 [77]. The two complexes have been proposed to work in opposite directions on tRNA genes, where Isw2 aligns the nucleosome towards the TSS and Isw1 pushes the nucleosome away from the gene body. This antagonistic action could then create a nucleosome free region with a strong positioned upstream nucleosome at RNAPIII promoters [77]. The role of Isw2 in tRNA transcription is still unknown, but it has been found that Isw2 plays a role in Ty1 integration site selection, which is linked to tRNA genes [108].

The chromatin remodeler CHD8 plays a role in RNAPIII transcription in human cells. In collaboration with the transcription factor ZNF143, CHD8 contributes to efficient transcription of the human U6 small nuclear RNA [110]. Additionally, the ISWI-containing complex B-WICH (related to the yeast RSC complex) remodels chromatin in the vicinity of 5S rRNA and 7SL genes and is required for proper assembly of RNAPIII on these genes by promoting binding of c-Myc [111].

### Histone chaperones and histone exchange

While chromatin remodelers target nucleosomes, histone chaperones handle non-nucleosomal histones to establish and ensure a correct chromatin structure required for transcription. Asf1 is a general histone chaperone that mediates eviction of nucleosomal histones and deposition of evicted and newly synthesized histone H3 during RNAPII elongation [112]. In yeast, Asf1 associates with highly transcribed RNAPIII genes [112,113]. Asf1 especially occupies the coding region of tRNA genes with a similar distribution as RNAPIII. In Asf1 knockout cells, nucleosome occupancy at Asf1 targets (both RNAPII- and RNAPIII-transcribed genes) is lower while tRNA levels are increased [113]. However, as Asf1 has many targets in the genome, further studies are needed to determine which of the transcriptional changes are direct versus indirect effects.

Another well-studied histone chaperone is the FACT complex. FACT has been proposed to aid RNAPII elongation by destabilization of nucleosomes through histone deposition and removal [114–116]. Yeast FACT consists of Spt16 and Pob3, while Nhp6 is responsible for loading FACT onto nucleosomes [104,115,117]. Together with the SWR1 chromatin remodeling complex, which exchanges histone H2A for H2A.Z, FACT regulates the presence of H2A.Z in tRNA-gene flanking nucleosomes [104]. However, genome-wide mapping of H2A.Z showed that tRNA genes represent H2A.Z-free regions and that the flanked uniformly positioned nucleosomes harbor a low H2A.Z content [118]. In repressive conditions, H2A.Z levels increase at the *SUP4* tRNA gene [104]. This is contrast with the *SNR6* gene, which shows decreased H2A.Z occupancy [119]. This discrepancy suggests that H2A.Z plays different roles at tRNA genes and the *SNR6* gene, the mechanisms and relevance of which need to be further elucidated.

Genome-wide studies have shown that FACT is enriched at 3′-ends of RNAPIII-transcribed genes which implies a possible role in downstream nucleosome positioning [117,120]. Binding of FACT is decreased significantly at the downstream region in repressive conditions [117]. This is in contrast with the previous finding that FACT and its loader Nhp6 have an inhibitory role [104]. The FACT subunit Spt16 shows differential binding to individual tRNA genes but the occupancy does not correlate with RNAPIII transcript levels [117]. In general, tRNA transcription is not affected in the temperature-sensitive *spt16-197* mutant, but transcription was increased for a few tRNA genes. In contrast, RNA levels of other RNAPIII-transcribed genes are decreased in the Spt16 mutant [117]. In human cells, FACT and the RNAPIII subunit POLR3E interact directly and FACT occupies RNAPIII-transcribed genes [121]. Down-regulation of FACT through RNA interference reduces transcription levels of several RNAPIII-transcribed genes, including tRNA^Tyr^ and 7SL RNA. Together, these findings suggests that FACT plays a role in nucleosome organization and transcription of tRNA genes in a gene-specific manner.

### Histone acetylation

Post-translational modifications of histones play an important role in recruitment of remodeling complexes, chromatin remodeling, and regulation of transcription factor binding [122,123]. Acetylation of histone proteins is facilitated by HATs and is generally linked to more open chromatin structures and transcription activation. Histone deacetylases (HDACs) counteract the activity of HATs. Multiple HATs have been identified at tRNA genes, such as Gcn5 in human cells [124]. In budding yeast, HDACs Hda1 and Hos2 have been reported to bind tRNA genes [125]. Little is known about how HATs and HDACs are recruited to RNAPIII-transcribed genes and how chromatin acetylation and deacetylation affects RNAPIII activity. Recently, the HATs Gcn5 and Mst2 were found to occupy tRNA genes in fission yeast [126]. Gcn5 and Mst2 are recruited to tRNA genes through antisense RNAPII transcription (also discussed below). These two HATs synergize to maintain a high level of acetylation and low level of nucleosome occupancy at tRNA genes, which promotes high levels of chromatin-bound RNAPIII [126]. A recent study showed that the dynamics of histone acetylation can vary for different lysines on the tails of histone H3 and H4, with some acetylations showing an increase during conditions of repressed transcription (Figure 2) [127]. In contrast, several acetylation marks of the tail of histone H3 in the human genome were found to correlate positively with tRNA gene activity [128].

### Histone methylation

Histone lysine methylation marks both active (H3K4, H3K36 and H3K79) and repressed (H3K9, H3K27 and H4K20) transcription [129]. Genome-wide profiling of epigenetic features of RNAPIII-transcribed genes in the human genome showed that marks associated with active RNAPII transcription, such as H3K4me1/2/3, are present at most but not all RNAPIII genes, while H3K27me3 showed the opposite pattern (Figure 2) [128]. By using RNAPIII occupancy as a proxy for transcriptional output, H3K4 methylation correlates with tRNA expression and H3K27 methylation with repression, similar to RNAPII-transcribed genes [128]. Methylation of H3K9 has also been linked to suppression of RNAPIII-mediated transcription of SINE elements in the mammalian genome [130]. The repressive mark H3K27me3 is mediated by EZH2, a member of the Polycomb repression complex PRC2. EZH2 has been reported to interact with TFIIIC and is especially enriched at inactive tRNA genes [131]. This suggests that EZH2 deposits H3K27me3 at RNAPIII-transcribed genes through TFIIIC. This process also requires the PRC2 component SUZ12 [131]. Polycomb group proteins are epigenetic regulators that play a key role in development and differentiation [132]. In the process of development, it is known that specific subsets of tRNA genes become active or silenced [30,133]. Possibly, tRNA genes are inactivated during development in a process that involves Polycomb to deposit repressive epigenetic marks.

### DNA methylation

DNA methylation is an epigenetic mark which regulates gene expression [134]. In an *in vitro* system using *Xenopus* oocyte extracts, DNA methylation was found to inhibit transcription of tRNA genes [135]. Corroborating this finding, TFIIIC has been reported to preferentially recognize non-methylated DNA and to be excluded by CpG methylation [136] and ablation of the activity of the DNA methyl transferases DNMT1 and DNMT3 increased transcription of the U6 snRNA gene family [137]. Furthermore, CpG methylation and RNAPIII occupancy are anti-correlated in human mammary epithelial cells [138]. Variations in DNA methylation at tRNA genes was furthermore found to be associated with specific alterations of tRNA expression in cancer cells [139]. Together, these findings suggest that DNA methylation has a negative role in transcription of RNAPIII-transcribed genes.

### Chromatin-binding proteins

Besides the general RNAPIII transcription factors TFIIIB and TFIIIC, nucleosomes and chromatin modifiers and remodelers, other chromatin-binding proteins have been described to play a role in RNAPIII regulation. Sub1 (yeast homologue of PC4) is a transcriptional regulator and involved in activation, elongation and mRNA processing [140]. Genome-wide mapping of Sub1 showed that it is present on RNAPIII-transcribed genes [141]. Both in an *in vitro* reconstituted system and *in vivo*, Sub1 stimulates tRNA gene expression. Additionally, TFIIIB binding is decreased in *sub1Δ* yeast strains while TFIIIC remains unchanged [141]. Thus, Sub1 might promote RNAPIII transcription by facilitating TFIIIB recruitment by TFIIIC. The human homologue PC4 was shown to enhance and extend TFIIIC interaction with promoter and terminator sequences, strongly stimulating multiple-round transcription (and reinitiation) *in vitro* [142]. It remains to be determined whether PC4 has a similar role as Sub1 in RNAPIII transcription *in vivo.* Another candidate stimulator of RNAPIII transcription is Nab2, an RNA-binding protein that promotes 3′-end formation and mRNA export [143]. Nab2 occupies RNAPIII-transcribed genes genome-wide and Nab2 recruitment depends on active RNAPIII transcription [144,145]. In addition, full occupancy of RNAPIII requires Nab2. Nab2 is proposed to function through TFIIIB, as TFIIIB occupancy decreases in Nab2 deficient yeast cells while TFIIIC remains unaffected [144,145]. Similarly, the human La protein is found to occupy RNAPIII-transcribed genes independent of RNAPIII [146]. La is suggested to function in both transcript release and reinitiation by RNAPIII [147–149].

**Figure 3.**
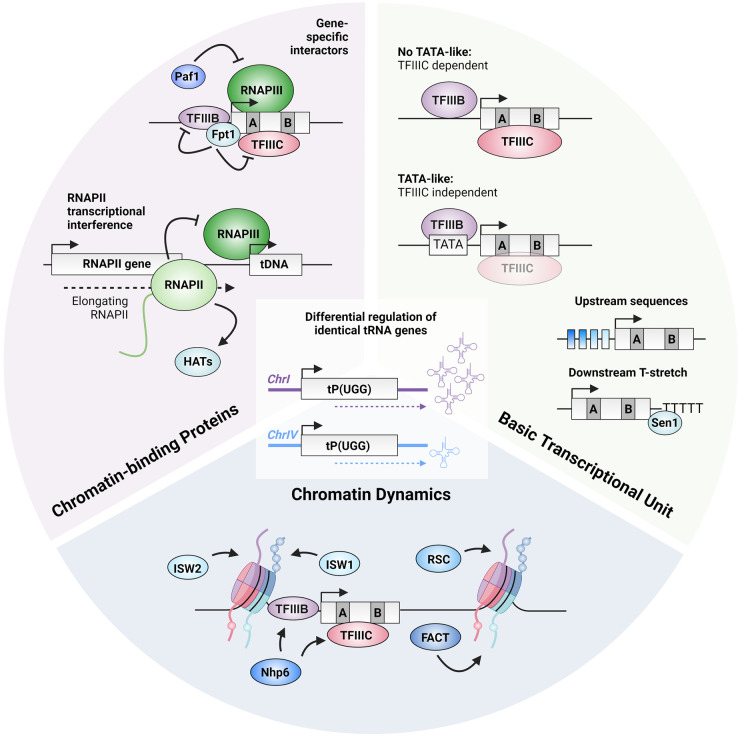
Cellular contributors to gene-specific regulation of tRNA genes. tRNA genes can be differentially regulated in a gene-specific manner, even when they have identical body sequences and hence identical gene regulatory sequences [18,19,27–30]. From a basic transcriptional perspective, the upstream and downstream sequences could contribute to this observation Based on *in vitro* data, the presence or absence of an upstream TATA-like element could hypothetically reflect a weaker or stronger TFIIIC-dependency, respectively [52]. Variations in conserved upstream sequences and strength of downstream terminator sequences, combined with accessory proteins such as Sen1, could also contribute to differential regulation. On the level of chromatin dynamics, differential chromatin remodeling could contribute to differential regulation of tRNA genes. The nucleosome remodeler Nhp6 stabilizes the interaction of TFIIIC with the A- and B-box and ensures correct TFIIIB placement. Suboptimal upstream TFIIIB binding sites affect Nhp6 dependency. Both RSC and FACT are suggested to play a role in maintaining a nucleosome-free region downstream of the TSS of tRNA genes. RSC occupancy varies across tRNA genes and FACT affects transcription in a gene-specific manner. Upstream of the TSS, ISW1 and ISW2 work antagonistically to ensure a strongly placed upstream nucleosome. Additionally, histone modifications such as acetylation show different dynamics flanking identical tRNA genes. Several chromatin-binding proteins have been suggested to affect differential tRNA gene regulation. RNAPII transcription can on the one hand interfere with RNAPIII in a gene-specific manner and on the other hand promote the nucleosome free state and thereby RNAPIII transcription. This takes place by recruitment of HATs by RNAPII, potentially affecting each tRNA gene in a context-dependent manner. Finally, several tRNA gene interactors have been described to have a non-uniform repressive role in RNAPIII assembly at tRNA genes, such as Fpt1 and Paf1.

Most chromatin-associated factors, histone modifiers, and chromatin remodelers associated with regulation of tRNA genes bind at many places in the genome and are not specific to tRNA genes. Therefore, it often remains challenging to distinguish between direct from indirect effects in the examples discussed above. A further challenge is the notion that within the group of tRNA genes, differential regulation of identical tRNA genes has been observed, indicating that information on the level of individual tRNA genes is crucial to understand how chromatin processes affect tRNA gene regulation (Figure 3). Analysis of engaged RNAPIII on chromatin in budding yeast has shown that tRNA genes with identical gene bodies and hence identical regulatory sequences respond differently to stress signals [19]. tRNA genes that respond poorly to repressive signals have been classified as ‘housekeeping’, while down-regulated tRNA genes have been classified as ‘regulated’. A recent study identified the previously uncharacterized protein Fpt1 as a factor that occupies all tRNA genes in yeast [73]. Fpt1 uniquely but differentially occupies all tRNA genes and shows higher occupancy at regulated tRNA genes than at housekeeping genes, while RNAPIII levels do not much differ between tRNA genes in active conditions. In the absence of Fpt1, the eviction of RNAPIII from tRNA genes upon exposure to nutrient stress is compromised, suggesting that Fpt1 is a negative regulator of RNAPIII assembly at regulated tRNA genes. Acute protein depletion experiments indicate that Fpt1 is recruited via TFIIIB and TFIIIC and does not require transcription or RNAPIII [73]. This indicates that Fpt1 might affect RNAPIII at tRNA genes through interactions with the general transcription factors. Another factor that shows differential binding to tRNA genes is the Paf1 complex, also involved in transcription elongation of RNAPII (and thus not a unique regulator of RNAPIII) [150]. Paf1 occupancy at tRNA genes is low and does not correlate with RNAPIII occupancy. However, Paf1 deletion causes increased occupancy of RNAPIII in a gene-specific manner. Further studies are necessary to determine whether Paf1 has a direct role in RNAPIII transcription.

## The genomic context of tRNA genes

### Short distance: neighboring genes and RNAPII

Above, we discussed the role of internal and flanking DNA sequences and chromatin-associated factors in RNAPIII regulation. These examples focused mainly on what takes place on and immediately next to tRNA genes. In contrast with regulation of RNAPII genes, no tRNA-gene specific regulators have been described that directly act on RNAPIII. One emerging mechanism by which genomic context can regulate tRNA genes differently across the genome is by transcriptional interference from neighboring RNAPII-transcribed genes, and RNAPII itself. In *Arabidopsis thaliana*, transcription by RNAPII and RNAPIII of target genes encoded on opposing strands in the same DNA window is anti-correlated [151]. Similarly, the RNAPIII transcribed *MIR* element within the first intron of the *Polr3e* gene, transcribed by RNAPII, contributes to regulation of *Polr3e* expression [152]. This finding has been further corroborated in human cells [74] and mouse embryonic stem cells [153]. Here, rapid depletion of RNAPII subunit Rpb1 via an auxin-inducible degron, caused up-regulation or RNAPIII occupancy at the *MIR* element. Additionally, RNAPII depletion caused up-regulation of specific tRNAs. This suggests that RNAPII transcription can interfere with RNAPIII in a gene-specific manner in mammals [74,153].

RNAPII itself is also found in close proximity to tRNA genes in human and mouse cells [12–14,128,154,155]. For example, RNAPII occupies the 300 bp upstream region of the five active human U6 genes [155]. The drug α-amanitin, used to inhibit RNAPII specifically, causes a reduction in U6 snRNA levels, suggesting a role for RNAPII in RNAPIII transcription [155]. However, this effect is likely at least in part indirect as α-amanitin treatment also affects transcription of c-Myc, a known RNAPIII activator [14,74,156]. Hence, RNAPII can regulate RNAPIII directly or indirectly by regulating expression of c-Myc. With regard to direct positive effects it has been suggested that RNAPII activity is required to remodel the chromatin environment of tRNA genes [12,153]. RNAPII, chromatin modifications associated with active RNAPII, and RNAPIII occupancy are correlated and tRNA genes unoccupied by RNAPIII also lack RNAPII or active chromatin [12]. This result is corroborated by recent findings in fission yeast showing that RNAPII is required to maintain an open chromatin state at tRNA genes [126]. Specifically, the authors found that antisense RNAPII transcription from downstream of the tRNA genes leads to recruitment of two HATs, Gcn5 through the SAGA complex, and Mst2 via the C-terminal domain of RNAPII. Together, these factors are required to maintain a nucleosome-depleted state at tRNA genes and prime them for activation and expression by RNAPIII [126]. These findings suggest that the open chromatin state of each tRNA gene can be affected by its genomic location, specifically its proximity to a TSS of an RNAPII transcribed gene, which opens up opportunities for tRNA-gene specific regulation by transcription factors that regulate RNAPII. Taken together, RNAPII is proposed to have two opposing effects on RNAPIII transcription. On the one hand, RNAPII interferes with RNAPIII through transcriptional interference, on the other hand, antisense RNAPII transcription primes an open chromatin state for efficient RNAPIII transcription through recruitment of HATs.

In addition to RNAPII itself, the mammalian RNAPII transcription factors AP-1 (c-Fos, c-Jun) and c-Myc have also been associated with RNAPIII-transcribed genes [14]. c-Myc binds to TFIIIB to directly activate RNAPIII transcription [156]. Activation by c-Myc is controlled by HATs GCN5 and cofactor TTRAP [124]. Repression of RNAPIII is facilitated by the tumor suppressor p53 through interaction with TFIIIB and TBP [157] and the Retinoblastoma tumor suppressor protein, Rb, through direct interactions with TFIIIB (as reviewed in [158]).

### Long distance: 3D genome organization

Chromatin organization in the 3D nuclear space is important for regulation of gene expression [159]. *In situ* fluorescent hybridization in budding yeast has shown that tRNA genes localize near the periphery of the nucleolus. Inactive tRNA genes cluster less near the nucleolus, which suggests that clustering of tRNA genes increases TFIIIB, TFIIIC and RNAPIII concentration for efficient transcription or that clustering is a consequence of transcription [160,161]. Hi-C (high-throughput analysis of higher-order chromatin structure) also showed an association among 64 tRNA genes. However, the authors emphasize that the association between tRNA genes is biased by the genomic distance of the tRNA genes from the centromere, indicating that the perceived associations between tRNA genes might be related to centromere clustering and hence not reflect functional organization of tRNA genes themselves [162]. To solve the discrepancy between the Hi-C data [162] and fluorescence microscopy data [161], a genome simulation model was used to determine the spatial arrangements of tRNA genes in budding yeast. The majority of tRNA genes are distributed around centromeres (58%), while a smaller fraction distributed around rDNA regions. Integration of tRNA sequencing data in the model shows that transcriptional levels do not correlate with nuclear localization [163]. Instead, topological organization of tRNA genes might have roles in genome organization by the insulator activity of tRNA genes, which is described in several other reviews [95,164–166]. However, the role of tRNA genes in 3D genome organization was recently challenged by the analysis of yeasts with a synthetic genome in which the tRNA genes were rearranged, with little impact on 3D genome organization [167].

## Perspectives

- Transcription of tRNA genes by RNAPIII has long been considered a default state, regulated by general factors and pathways. However, it is clear that tRNA genes are non-uniformly regulated in the context of disease and development.
- In a wide range of models ranging from yeast, flies and plants to mammals, it has become clear that each tRNA gene has its own specific behavior and regulation.
- Studies on the genomic context of tRNA genes and the differential occupancy and dependencies on chromatin factors start to provide a rich source of leads to untangle the gene-specific regulation of tRNA genes in health and disease.

## Abbreviations

DSE: distal sequence element
HAT: histone acetyltransferase
HDAC: histone deacetylase
ICR: internal control region
PSE: proximal sequence element
RNAPIII: RNA polymerase III
RNAPII: RNA polymerase II
SINE: Short Interspersed Nuclear Element
TBP: TATA-binding protein
TSS: transcription start site
